# COVID-19 vaccine response and safety in patients with cancer: An overview of systematic reviews

**DOI:** 10.3389/fpubh.2022.1072137

**Published:** 2022-11-15

**Authors:** Hong Sun, Fengjiao Bu, Ling Li, Xiuwen Zhang, Jingchao Yan, Taomin Huang

**Affiliations:** Department of Pharmacy, Eye and ENT Hospital, Fudan University, Shanghai, China

**Keywords:** COVID-19, vaccine, cancer, response, safety

## Abstract

**Background:**

To date, the COVID-19 pandemic does not appear to be overcome with new variants continuously emerging. The vaccination against COVID-19 has been the trend, but there are multiple systematic reviews on COVID-19 vaccines in patients with cancer, resulting in redundant and sub-optimal systematic reviews. There are still some doubts about efficacy and safety of the COVID-19 vaccine in cancer patients.

**Purpose:**

To identify, summarize and synthesize the available evidence of systematic reviews on response and COVID-19 vaccine safety in patients with cancer.

**Methods:**

Multiple databases were searched from their inception to May 1, 2022 to fetch the relevant articles. Study quality was assessed by AMSTAR2. The protocol of this study was registered on PROSPERO (CRD42022327931).

**Results:**

A total of 18 articles were finally included. The seroconversion rates after first dose were ranged from 37.30–54.20% in all cancers, 49.60–62.00% in solid cancers and 33.30–56.00% in hematological malignancies. The seroconversion rates after second dose were ranged from 65.30–87.70% in all cancers, 91.60–96.00% in solid cancers and 58.00–72.60% in hematological malignancies. Cancer types and types of therapy could influence vaccine response. COVID-19 vaccines were safe and well–tolerated.

**Conclusions:**

This study suggests COVID-19 vaccine response is significantly lower in cancer patients. Number of received doses, cancer types and treatment strategies could influence response of COVID-19 vaccine in cancer patients. COVID-19 vaccines are safe and well–tolerated. Considering the emergence of several new variants of SARS-CoV-2 with potential influence on ongoing vaccination programs, there is a need for booster doses to increase the effectiveness of COVID-19 vaccines.

**Systematic review registration:**

https://www.crd.york.ac.uk/prospero/display_record.php?ID=CRD42022327931, identifier CRD42022327931.

## Introduction

Coronavirus disease 2019 (COVID-19), which is caused by SARS-CoV-2, has caused significant discomfort and death worldwide (1, 2). Globally, by 21 July 2022, more than 560 million COVID-19 cases were confirmed, and 6.37 million deaths were reported worldwide (3). There is substantial evidence that cancer patients are placed in a vulnerable state and the risk of death from COVID-19 is higher (4–6). To date, the COVID-19 pandemic does not appear to be overcome with the continuously emerging new variants (7, 8) and the burden of COVID-19 related morbidity and mortality in patients with cancer is still significant.

**Figure 1:**
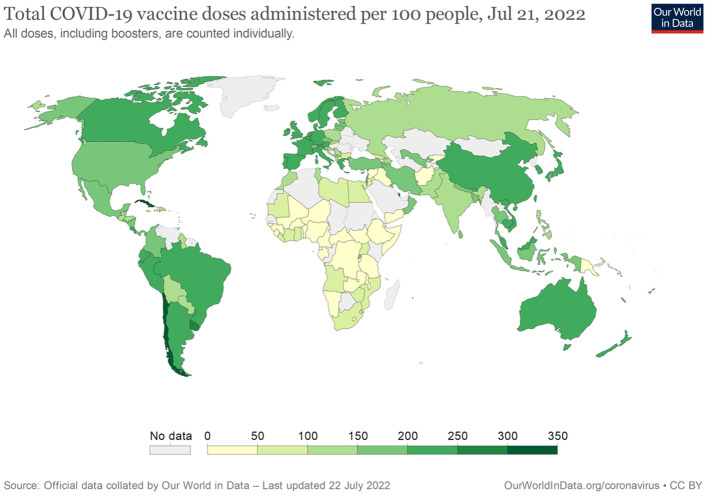
The distribution of total number of people who have been fully vaccinated against COVID-19.

Despite established supportive therapies and new approved antiviral drugs, the COVID-19 vaccines emerged as the primary strategy fighting against COVID-19 pandemic. COVID-19 vaccines were rapidly developed with 172 vaccines in clinical development and 199 in pre-clinical stage at the time of writing (9). Several COVID-19 vaccines such as mRNA-1273 (Moderna) and BNT162b2 (Pfizer-BioNTech), displayed efficacy and safety in the large phase II and III clinical trials and obtained the emergency approval by the regulatory agencies (10, 11). As of April 8, 2022, several vaccines against COVID-19 assessed by WHO have met necessary criteria for efficacy and safety. There are some differences among these COVID-19 vaccines. At first, the vaccine types are different. ChAdOx1-S [recombinant] vaccine (AstraZeneca), Ad26.COV2.S vaccine (Johnson & Johnson) and Ad5-nCoV-S [recombinant] vaccine (CanSinoBio) are developed based on viral vector. NVX-CoV2373 (Novavax) is based on protein subunit. Vaccines based on mRNA include mRNA-1273 (Moderna) and BNT162b2 (Pfizer-BioNTech). COVID-19 vaccine BIBP (Sinopharm), CoronaVac (Sinovac) and BBV152 COVAXIN vaccine (Bharat Biotech) are based on inactivated viruses. Secondly, the recommended dosage and interval are various. In addition to CanSinoBio, Johnson & Johnson and Pfizer-BioNTech vaccines, the recommended dosage of other vaccines is two doses. The WHO Strategic Advisory Group of Experts on Immunization (SAGE) recommends the use of CanSinoBio vaccine as a single dose. A single dose regimen of Johnson & Johnson vaccine remains an acceptable option. However, WHO recommends all efforts should be taken to provide two doses of this vaccine. For persons aged 5 years and above, the recommended dosage of Pfizer-BioNTech vaccine is two doses, while for children aged 6 months to 4 years, the recommended schedule is three doses. The interval of majority vaccines between the first and second dose is 4–8 weeks. Furthermore, age group for vaccination is different. Most vaccines are authorized for use for individuals aged 18 years and above except for Moderna and Pfizer-BioNTech for those aged 6 months and above, Novavax for those aged 12 years and above. The detailed information about these COVID-19 vaccines were shown in Supplementary Table 1. Almost all COVID-19 vaccines have shown remarkable efficacy and safety in the general population and have decreased COVID-19 related-mortality and morbidity worldwide. To date, more than 12 billion vaccine doses have been administered (Figure 1) (12). These vulnerable populations should be prioritized for the vaccination against SARS-CoV-2 due to the higher rate of morbidity and mortality of COVID-19 in patients with cancer. However, the available data are limited among cancer patients because of their ineligibility in most clinical trials. Although vaccination against SARS-CoV-2 is recommended for cancer patients as long as no contraindications to any component of COVID-19 vaccines. Worryingly, the results of COVID-19 vaccination are considered insufficient in patients with cancer, especially when patients with hematological malignancies (HM) on anti-CD20 therapy (13).

It is significant to understand the efficacy and safety of COVID-19 vaccines in cancer patients because of the lack of effective treatments for COVID-19. Currently, the literature encompasses multiple systematic reviews on COVID-19 vaccines in patients with cancer, resulting in redundant and sub-optimal systematic reviews. Better understanding of overall efficacy and safety of COVID-19 vaccines in patients with cancer could protect the vulnerable populations. This overview of systematic reviews and meta-analyses aims to evaluate the current available evidence on the efficacy and safety of COVID-19 vaccines in cancer patients.

## Methods

This study was conducted in accordance with the PRISMA (Preferred Reporting Items for Systematic Reviews and Meta-Analyses) checklist (14). A protocol was registered with PROSPERO (CRD42022327931).

### Search strategy

Multiple databases were searched from their inception to May 1, 2022 by two reviewers independently: PubMed, Cochrane Library, Web of Science, EMBASE, and CNKI databases. Keywords for searching included “COVID”, “COVID-19”, “SARS-CoV-2”, “vaccine”, “vaccination”, “cancer”, “neoplasms”, etc., The detailed search strategy was listed in Supplementary Table 2. There was no restriction regarding the publication language.

### Eligibility criteria

We aimed to identify all systematic reviews with or without meta-analyses that summarized and reported the COVID-19 vaccine response or safety in cancer patients. Only published systematic reviews with or without meta-analyses, which clearly identified the response of COVID-19 vaccines in patients with cancer, as compared to a non-cancer group (if any), and which investigated any adverse events (AEs) of COVID-19 vaccines in cancer patients were included. Systematic reviews and meta-analyses were eligible. The exclusion criteria were studies with different study designs such as case reports, editorial letters, narrative review articles, randomized controlled trials (RCTs), observational studies, opinion papers and animal studies.

### Study selection and data extraction

According to the PRISMA guidelines, data extraction was performed and independently verified by two authors. Extraction data included: first author; publication year; search details; number of included studies; assessment of risk of bias and/or study quality; outcome investigated; COVID-19 vaccine types; cancer diagnoses; results. Discrepancies were resolved through discussion.

### Study quality assessment

The methodological quality of included systematic reviews was evaluated by the Assessing the Methodological Quality of Systematic Reviews (AMSTAR 2) which consists of 16 items (15). Based on the weaknesses in critical domains, the AMSTAR 2 assessment could generate an overall quality rating. According to the quality rating confidence levels, quality of systematic reviews was rated as “high”, “moderate”, “low” and even “critically low”. Two authors independently evaluated all systematic reviews to ensure interrater reliability. Disagreements among authors were solved by consensus with involvement of another author. The interrater reliability of quality assessment was assessed by Kappa coefficient.

## Results

### Search results

**Figure 2:**
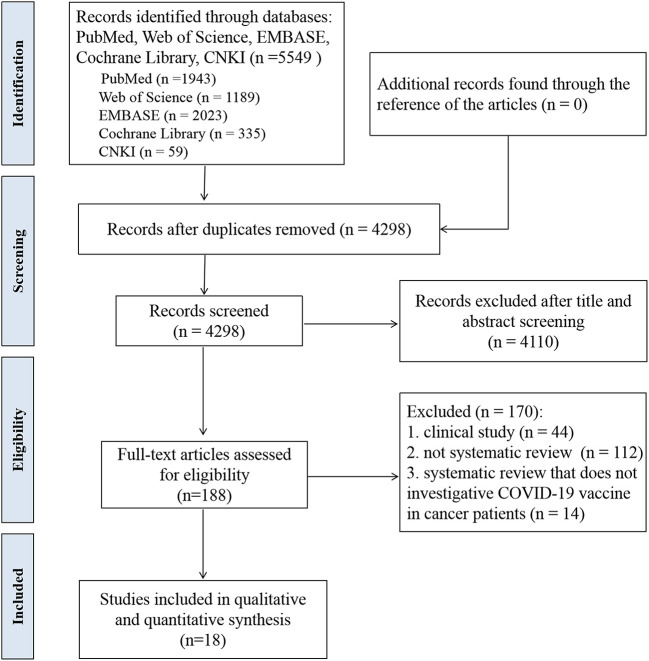
PRISMA flowchart of the identification and inclusion of systematic reviews.

A total of 5,549 records were identified throughout database search. Records were 4,298 after the removal of the duplicate. By title/abstract screening, 188 articles were identified for full-text view. Finally, 18 systematic reviews (15 with meta-analyses and 3 without meta-analyses) were identified which fulfilled the eligibility criteria (16–33). The study flow was depicted according to the PRISMA guidelines in Figure 2.

### Characteristics of the included studies

The details of the included studies were summarized in Supplementary Table 3. These systematic reviews were published between 2021 and 2022. The total number of original studies included in these systematic reviews ranged from 5 to 64. All systematic reviews included only observational studies, without RCTs. The majority of systematic reviews (15/18, 83.30%) included a control group and three systematic reviews included only patients with cancer.

### Quality assessment of included studies

**Figure 3:**
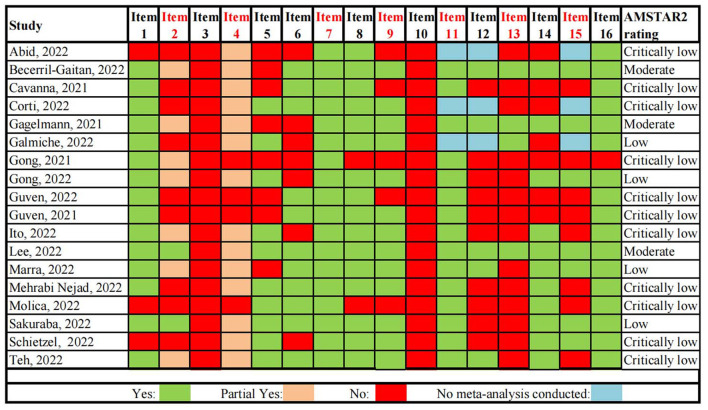
Quality assessment of included systematic reviews based on AMSTAR-2. Critical domains are highlighted in red.

Methodological quality of 18 eligible systematic reviews was evaluated by the AMSTAR 2. The results of AMSTAR 2 were summarized in Figure 3. The confidence levels of results of the included systematic reviews were moderate in three, low in four and critically low in eleven. The result of interrater reliability suggested good results (percentage agreement 90.28% and Cohens'k = 0.84). None of the systematic reviews met the criteria of Item 3 and Item 10. The failure to take these two items into account, no systematic review was rated with high confidence. The study selection and data extraction of systematic reviews were not carried out independently by two reviewers, resulting in one systematic review being rated with moderate confidence. Another systematic review was also rated as moderate with no discussion and explanation of the heterogeneity in the results and lack of study selection in duplicate. The majority of systematic reviews were lack of a study protocol which should be established prior to conduct of the systematic review. Some studies didn't take the risk of bias (RoB) into account. And some studies did not adequately investigate publication bias (small study bias) and did not discuss its possible impact on review results.

### COVID-19 vaccine response

Overall, COVID-19 vaccine response was significantly lower in cancer patients. The number of received doses, cancer types and types of therapy could influence the response of COVID-19 vaccine in cancer patients.

### Response after the first dose of COVID-19 vaccine

**Figure 4:**
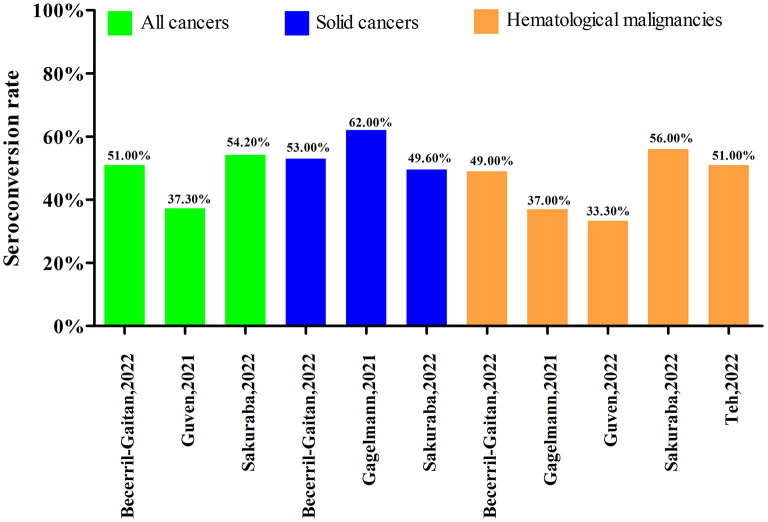
Seroconversion rate after the first dose of COVID-19 vaccine.

There were six systematic reviews with meta-analyses that assessed the seroconversion rate after first dose of COVID-19 vaccine in cancer patients. The studies by Becerril-Gaitan et al. (17), Gagelmann et al. (20), and Sakuraba et al. (31) reported outcomes both in solid cancers and hematological malignancies. The studies by Guven et al. (24) and Teh et al. (33) reported outcomes only in hematological malignancies. The study by Guven et al. (25) reported outcomes in all cancers without subgroup analysis by cancer type. After first dose of COVID-19 vaccine, the low seroconversion rates were consistent across all studies. As shown in Figure 4, the seroconversion rates after first dose of COVID-19 vaccine in cancer patients were, respectively 37.30, 51.00, and 54.20% in three systematic reviews with meta-analyses. The seroconversion rates were ranged from 49.60 to 62.00% in solid cancers and 33.30 to 56.00% in hematological malignancies. In addition, the seroconversion rate after first dose of COVID-19 vaccine in a systematic review without meta-analysis ranged widely from 11.00 to 87.50% in all cancers, 25.00 to 67.00% in solid cancers and 11.00 to 87.50% in hematological malignancies (19).

**Figure 5:**
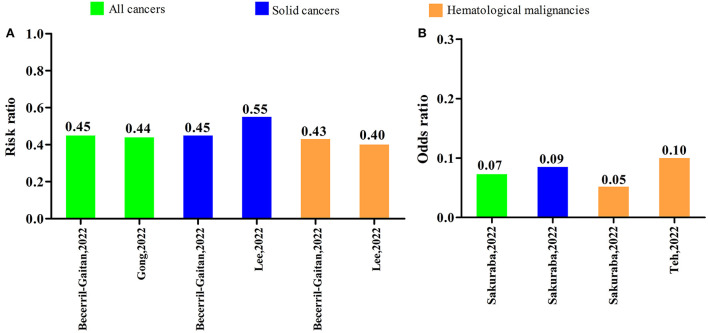
Serologic response after the first dose of COVID-19 vaccine compared to controls. **(A)** Risk ratio **(B)** Odds ratio.

Five systematic reviews with meta-analyses reported on comparison of the serologic response after first COVID-19 vaccine dose to controls. Three systematic reviews with meta-analyses reported risk ratios (RRs) and two others reported odds ratios (ORs). The RRs were 0.44 [95% confidence interval (CI): 0.26–0.73] and 0.45 (95% CI: 0.35–0.58) in all cancers, 0.45 (95% CI: 0.37–0.55) and 0.55 (95% CI: 0.46–0.65) in solid cancers, 0.40 (95% CI: 0.32–0.50) and 0.43 (95% CI: 0.29–0.63) in hematological malignancies. The ORs were 0.07 (95% CI: 0.03–0.20) in all cancers, 0.09 (95% CI: 0.02–0.29) in solid cancers, 0.05 (95% CI: 0.01–0.33), and 0.10 (95% CI: 0.04–0.29) in hematological malignancies. These results were shown in Figure 5.

### Response after the second dose of COVID-19 vaccine

**Figure 6:**
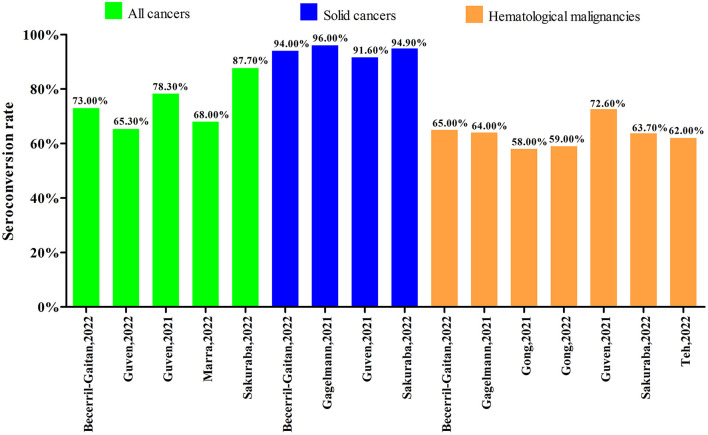
Seroconversion rate after the second dose of COVID-19 vaccine.

Nine systematic reviews with meta-analyses focused on the seroconversion rate after the second dose of COVID-19 vaccine. Among them, five systematic reviews with meta-analyses reported the outcomes in all cancers, four in solid cancers and seven in hematological malignancies. As shown in Figure 6, the seroconversion rates after the second dose of COVID-19 vaccine were ranged from 65.30 to 87.70% in all cancers, 91.60 to 96.00% in solid cancers, and 58.00% to 72.60% in hematological malignancies. Two systematic reviews without meta-analyses also reported the data after second dose of COVID-19 vaccine. The systematic review by Corti et al. (19) reported that the seroconversion rate ranged from 7.30 to 100.00% in all cancers, from 47.50 to 100.00% for solid cancers and from 7.30 to 88.80% for hematological malignancies. The seroconversion rates among all cancers, solid cancers and hematological malignancies in the systematic review by Galmiche et al. (21) were ranged from 39.00 to 98.00%, 64.00 to 98.00%, and 39.00 to 86.00%, respectively.

**Figure 7:**
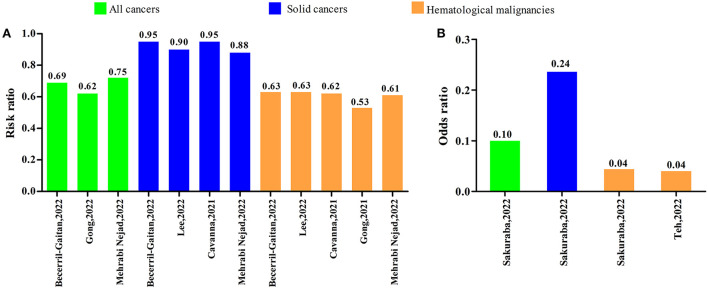
Serologic response after the second dose of COVID-19 vaccine compared to controls. **(A)** Risk ratio **(B)** Odds ratio.

Eight systematic reviews with meta-analyses investigated comparison of the serologic response after second COVID-19 vaccine dose to controls. Among the eight studies, six systematic reviews with meta-analyses reported RRs and the others reported ORs. As shown in Figure 7A, the RRs were ranged from 0.62 to 0.75 in all cancers, 0.88 to 0.95 in solid cancers, and 0.53 to 0.63 in hematological malignancies. The ORs were 0.10 (95% CI: 0.04–0.26) in all cancers, 0.24 (95% CI: 0.06–0.90) in solid cancers, 0.04 (95% CI: 0.01–0.16), and 0.04 (95% CI: 0.02–0.08) in hematological malignancies (Figure 7B).

### Subgroup analysis according to cancer types

**Figure 8:**
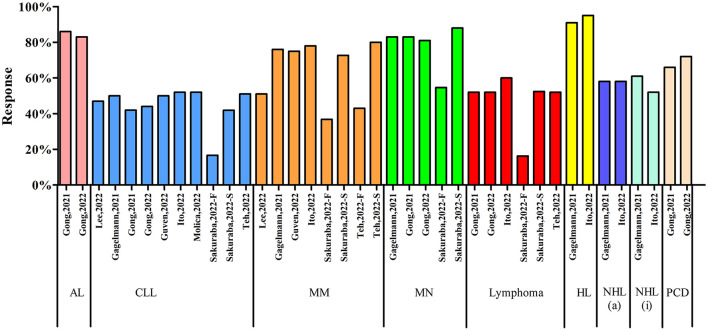
Subgroup analysis according to cancer types. AL, acute leukemia; CLL, chronic lymphocytic leukemia; MM, multiple myeloma; MN, myeloproliferative neoplasms; HL, Hodgkin's lymphoma; NHL(a), aggressive non-Hodgkin's lymphoma; NHL(i), indolent non-Hodgkin's lymphoma; PCD, plasma cell dyscrasias.

Nine systematic reviews with meta-analyses reported the subgroup analysis according to cancer types (Figure 8). The serologic response in acute leukemia (AL) reported in two systematic reviews were 86.00 and 83.00% (RR = 0.82), respectively. There were nine systematic reviews with meta-analyses reported the serologic response in chronic lymphocytic leukemia (CLL) and the outcomes were ranged from 16.70 to 52.00%. Among the nine articles, Sakuraba et al. reported both the results after first dose and second dose of COVID-19 vaccines (31). The vaccine response after the first dose was only 16.70% while the response was 41.90% after the second dose. Six systematic reviews with meta-analyses reported the vaccine response in multiple myeloma (MM). Two articles reported both the results after the first dose and second dose of COVID-19 vaccines. Sakuraba et al. reported that the response after the first dose and second dose of COVID-19 vaccine were 36.80% and 72.70%, respectively (31). Teh et al. reported the vaccine response was 43.00% after first dose and 80.00% after second dose (33). The results in the other four articles were ranged from 51.00 to 78.00%. Four systematic reviews with meta-analyses reported the vaccine response in myeloproliferative neoplasms (MN) and the results were ranged from 81.00 to 88.00%. Five articles focused on the lymphoma, the vaccine responses were over 50.00% (52.00 to 60.00%), except one first dose result was 16.30%. Gagelmann et al. (20) and Ito et al. (26) reported the vaccine response in Hodgkin's lymphoma, aggressive non-Hodgkin's lymphoma and indolent non-Hodgkin's lymphoma. The results were 91.00 and 95.00% (RR = 0.95) in Hodgkin's lymphoma, 58.00 and 58.00% (RR = 0.60) in aggressive non-Hodgkin's lymphoma, 61.00 and 52.00% (RR = 0.54) in indolent non-Hodgkin's lymphoma. Two articles reported the vaccine response in plasma cell dyscrasias and the results were 66.00% (RR = 0.73) and 72.00% (RR = 0.81). In addition, only one article reported the vaccine response in thoracic cancers, skin cancers, women's cancers, gastrointestinal cancers, urological cancers and brain cancers. The vaccine response in these solid cancers were ranged from 21.40 to 66.70% after first dose and 76.60 to 95.00% after second dose.

### Subgroup analysis according to types of therapy

**Figure 9:**
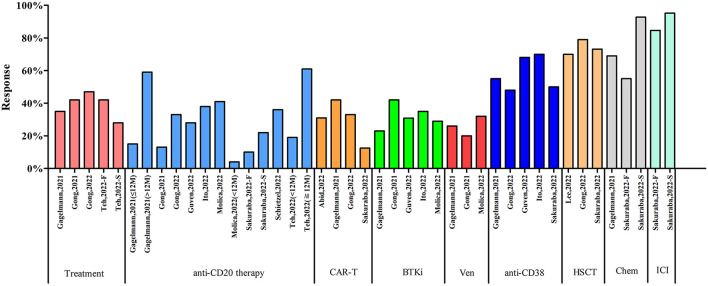
Subgroup analysis according to types of therapy. CAR-T, chimeric antigen receptor T-cell; BTKi, bruton tyrosine kinase inhibitor; Ven, venetoclax; HSCT, hematopoietic stem cell transplantation; Chem, chemotherapy; ICI, immune check-point inhibitors.

Ten systematic reviews with meta-analyses reported the influence of different treatments on COVID-19 vaccine response (Figure 9). Four articles reported the vaccine response in patients receiving active treatment. Teh, et al. reported the vaccine response was 28.00% after first dose and 42.00% after second dose (33). The vaccine response of other 3 articles were ranged from 35.00 to 47.00%. Nine systematic reviews with meta-analyses focused on the vaccine response in patients with anti-CD20 therapy. The response rates were ranged from 4.00 to 61.00%. Three articles reported vaccine response in patients receiving CD-20 antibody therapy within 12 months and seroconversion rates were 4.00, 15.00, and 19.00%, respectively. Two articles reported vaccine response in patients receiving CD-20 antibody therapy over 12 months with seroconversion rates were 59.00 and 61.00%. Sakuraba, et al. reported that seroconversion rate was 10.00% after first dose and 22.90% after second dose in patients receiving CD-20 antibody therapy (31). Four articles reported vaccine response in the patients receiving chimeric antigen receptor T-cell (CAR-T) therapy with seroconversion rates ranged from 12.50 to 42.00%. Five articles reported the influence of bruton tyrosine kinase inhibitor (BTKi) on COVID-19 vaccine response with seroconversion rates ranged from 23.00 to 42.00%. Several articles also reported the influence of other treatments on COVID-19 vaccine response, such as venetoclax (3 articles, 20.00 to 32.00%), anti-CD38 therapy (5 articles, 48.00 to 70.00%), hematopoietic stem cell transplantation (HSCT, 3 articles, 70.00 to 79.00%), chemotherapy (two articles; one article: first dose 55.00%, second dose 92.80%; another article: 69.00%), immune check-point inhibitors (one article; first dose 84.60%, second dose 95.20%), hormonal therapy (one article, 99.00%), and protease inhibitors (one article, 92.90%).

### COVID-19 vaccine safety

**Figure 10:**
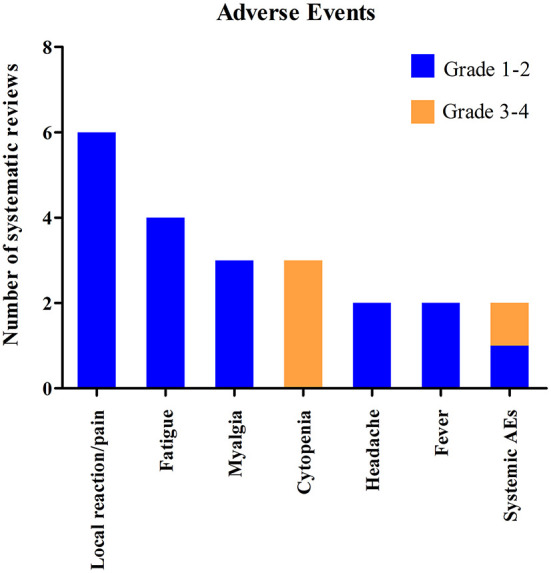
Systematic reviews included in the systematic review which investigated the adverse events of COVID-19 vaccines in cancer patients.

The safety of COVID-19 vaccines in cancer patients was investigated in six systematic reviews. Overall, the COVID-19 vaccines were reported to be safe and well–tolerated in these systematic reviews. Although three systematic reviews reported grade 3 AEs, they included one same study with four patients developed grade 3–4 cytopenia (34). The most commonly reported adverse events were local reaction, fatigue, myalgia, headache, fever, and cytopenia (Figure 10). The systematic review by Abid, et al. reported that the grade 3 or 4 cytopenia was in ~ 5% of entire cohort in one included study and lesser pain in CAR-T recipients compared to controls in another included study (16). Three studies included in the systematic review by Cavanna, et al. performed a safety analysis and reported that COVID-19 vaccines were generally very safe, with mostly mild or moderate adverse reactions reported (18). The systematic review by Corti, et al. reported that any-grade AEs ranged from 9.70 to 87.00% after first dose and from 23.00 to 85.00% after second dose of COVID-19 vaccines (19). The most commonly any-grade AEs were fatigue (first dose: range from 4.20 to 47.60%; second dose: range from 3.00 to 23.40%) and local pain (first dose: range from 7.40 to 69.00%; second dose: range from 32.30 to 67.20%). The systematic review by Gagelmann, et al. reported that the most frequent systemic AEs were generalized muscle pain (4.00–30.00%) and weakness/ fatigue (6.00–30.00%) (20). The systematic review by Gong et al. (22) also reported that the COVID-19 vaccines were generally well–tolerated with the most commonly AEs being local reaction, fever, headache, fatigue and myalgia. The systematic review by Teh et al. indicated that at least 1 AE rate was 39.00% after first dose and 36.00% after second dose (33). Local and systemic AEs of COVID-19 vaccines were mild except for one study with grade 3 systemic AEs rate from 1.00 to 2.00%.

## Discussion

COVID-19 vaccines have brought great hope to the whole world, although the end of the pandemic is still unclear. Patients with cancer are among the prioritized populations for the COVID-19 vaccination. However, data on the efficacy and safety of COVID-19 vaccines are limited in patients with cancer because of the exclusion of the vulnerable population from the clinical trials. Recently, there were several systematic reviews and meta-analyses on the COVID-19 vaccines in patients with cancer, resulting in redundant and sub-optimal systematic reviews. It is important to know the response and safety of COVID-19 vaccines in patients with cancer due to the lack of effective treatments for COVID-19. To our knowledge, this study is the first overview of systematic review and meta-analyses on response and safety of COVID-19 vaccines in cancer patients and provides a comprehensive summary of the currently available evidence on COVID-19 vaccine response and safety in patients with cancer. Although some overlaps existed among the included systematic reviews, these reviews still incorporated many different studies. The results demonstrated that cancer patients have a lower likelihood of COVID-19 vaccine response when compared with non-cancer controls. The responses of COVID-19 vaccine were especially lower in patients with hematologic malignancies which suggests an urgent need to improve the vaccination strategy in the vulnerable population.

The number of received doses could significantly influence the COVID-19 vaccine response. Patients with cancer have lower response rates after first dose of COVID-19 vaccine. Although the response rate was still lower than that of the controls, the rate was increased after second dose of COVID-19 vaccine, especially in patients with solid cancers (over 90.00% response rates). Cancer types could significantly influence the COVID-19 vaccine response. After second dose of COVID-19 vaccine, the patients with hematologic malignancies had a significantly lower response rate than those with solid cancers. We also performed subgroup analysis to investigate vaccine responses in different types of cancer. The subgroup analysis according to cancer types indicated that patients with CLL and non-Hodgkin's lymphoma have lower response rates compared to other types of cancers. In addition to the number of received dose and cancer types, types of therapy could also influence the response of COVID-19 vaccine. Patients with certain therapy have lower response rates of COVID-19 vaccine. Patients with active treatment have lower vaccine response rates. Additionally, anti-CD20, anti-CD38 therapy, BTKi, venetoclax and CAR-T therapy significantly decreased response rates. Furthermore, effect the of anti-CD20 therapy seemed to be long-lasting. Compare to patients received anti-CD20 therapy >12 months prior to vaccination, patientsreceived anti-CD20 antibody within 12 months prior to vaccination showed significantly reduced COVID-19 vaccine response. In contrast, therapies such as chemotherapy, hormonal therapy and immune check-point inhibitors had relatively higher response rates. Considering the limited studies on the influence of different therapies on COVID-19 vaccine responses among patients with cancer, further well-designed studies are warranted.

Due to properties of cancer and anti-cancer therapies, patients with cancer are immunocompromised and reported to have a greater COVID-19 related-mortality. The immune parameters such as B and T cell functions in patients with cancer might be changed by chemotherapy and patients with cancer have higher risks of various infections as well as reduced vaccine responses (35). Due to the lack of effective treatments of COVID-19, COVID-19 vaccine is vital for patients with cancer, although the vaccine response was lower. Patients with cancer should be encouraged to complete their COVID-19 vaccination schemes. Furthermore, several studies have indicated that there were some benefits with a booster vaccine dose after the completion of standard COVID-19 vaccination scheme among cancer patients. There was evidence that the vaccine efficacy against COVID-19 infection will decrease in time (36). Considering the emergence of several new variants of SARS-CoV-2 with potential influence on ongoing vaccination programs, there is a need for a booster dose to increase the effectiveness of COVID-19 vaccines (37). The booster doses of mRNA vaccines were reported to provide some protection against the omicron variant (38, 39) and severe COVID-19-related outcomes (40). Although several countries have removed the restrictions such as social distancing and use face masks for those individuals who have completed their COVID-19 vaccination schemes, these measures should not be applied to patients with cancer in whom COVID-19 vaccine response is significantly lower than that of general population.

Among all the included systematic reviews, there were only six articles reported the safety of COVID-19 vaccine in cancer patients (16, 18–20, 22, 33). Overall, the COVID-19 vaccines were reported to be safe and well–tolerated in these systematic reviews. The most commonly AEs were local reaction, fatigue, myalgia, headache, fever and cytopenia. Due to the limited studies on safety of COVID-19 vaccines in cancer patients, further studies on this issue are needed.

This systematic review has several limitations. Firstly, all systematic reviews included only observational studies, without RCTs. Many factors might influence the response to the COVID-19 vaccines, such as age and comorbidities (41, 42). These factors might not have been controlled for between the cancer group and non-cancer control group. Secondly, after a stringent quality assessment of the methods, most of the included systematic reviews did't reach an acceptable quality level. Higher quality evidence is needed to substantiate these findings. In addition, systematic reviews included in this study predominantly reported mRNA vaccines, subgroup analysis according to types of vaccines was limited. Different types of vaccines might have different efficacy and adverse effects (43). Moreover, there are some overlaps among the included systematic reviews with a same primary study in more than one systematic review. This could result in an overestimation of evidence. Furthermore, the types of immunoassay used in the included systematic reviews were not standardized. Lastly, despite we performed subgroup analyses according to cancer types and types of therapy, the number of articles reporting these detailed data was limited.

## Conclusions

In conclusion, the overview of systematic reviews demonstrates patients with cancer might have a lower COVID-19 vaccine response when compared with non-cancer controls, especially in patients with hematologic malignancies. The number of received doses, cancer types and types of therapy could influence the response of COVID-19 vaccine in patients with cancer. COVID-19 vaccines are reported to be safe and well–tolerated. There is a need for booster doses to increase the effectiveness of COVID-19 vaccines.

## Data availability statement

The original contributions presented in the study are included in the article/Supplementary material, further inquiries can be directed to the corresponding author/s.

## Author contributions

HS and TH conceived and designed the study. HS and FB conducted the database search, extracted the data, and data analysis. LL and XZ extracted the data. HS wrote the manuscript. TH and JY revised it critically for important intellectual content. All authors contributed to the article and approved the submitted version.

## Conflict of interest

The authors declare that the research was conducted in the absence of any commercial or financial relationships that could be construed as a potential conflict of interest.

## Publisher's note

All claims expressed in this article are solely those of the authors and do not necessarily represent those of their affiliated organizations, or those of the publisher, the editors and the reviewers. Any product that may be evaluated in this article, or claim that may be made by its manufacturer, is not guaranteed or endorsed by the publisher.
