# Supplementary material for: COVID-19 vaccine response and safety in patients with cancer: An overview of systematic reviews
Source: Front Public Health. 2022 Nov 15;10:1072137. doi: 10.3389/fpubh.2022.1072137 (PMC9707626; doi:10.3389/fpubh.2022.1072137)
Supplement: Supplementary file 1 [file Data_Sheet_1.docx]

# *Supplementary Material*

**Supplementary Table 1 The detailed information of COVID-19 vaccines**

| **Type of vaccine** | **Company** | **Vaccine** | **Age group for vaccination** | **Recommended dosage** | **Dose, interval and route of administration** |
| --- | --- | --- | --- | --- | --- |
| Viral vector | [AstraZeneca](https://www.who.int/news-room/feature-stories/detail/the-oxford-astrazeneca-covid-19-vaccine-what-you-need-to-know) | ChAdOx1-S [recombinant] vaccine | Aged 18 years and above | Two doses | 0.5 ml each dose, 8-12 weeks, intramuscular-deltoid muscle |
|  | [Johnson & Johnson](https://www.who.int/news-room/feature-stories/detail/the-j-j-covid-19-vaccine-what-you-need-to-know) | Ad26.COV2.S (COVID-19) vaccine | Aged 18 years and above | Single dose or two doses | 0.5 ml each dose, 2-6 months, intramuscular-deltoid muscle |
|  | [CanSinoBio](https://www.who.int/news-room/feature-stories/detail/the--cansino-biologics-ad5-ncov-s--recombinant---covid-19-vaccine--what-you-need-to-know) | Ad5-nCoV-S [recombinant] vaccine | Aged 18 years and above | Single dose | 0.5 ml, intramuscular-deltoid muscle |
| Protein subunit | [Novavax](https://www.who.int/news-room/feature-stories/detail/the-novavax-vaccine-against-covid-19-what-you-need-to-know) | NVX-CoV2373 | Aged 12 years and above | Two doses | 0.5 ml each dose, 8 weeks, intramuscular-deltoid muscle |
| mRNA | [Moderna](https://www.who.int/news-room/feature-stories/detail/the-moderna-covid-19-mrna-1273-vaccine-what-you-need-to-know) | mRNA-1273 | Aged 6 months and above | Two doses | Aged 17 and above: 100 µg, 0.5 ml each, 8 weeks apart; aged 12 to 17 years: 100 µg, 0.5 ml each, 4 weeks apart; aged 6 to 11 years: 50 µg,0.25 ml each, 4 weeks apart; aged 6 months to 5 years: 25 µg, 0.25 ml each, 4 weeks apart); intramuscular-deltoid muscle |
|  | [Pfizer/BionTech](https://www.who.int/news-room/feature-stories/detail/who-can-take-the-pfizer-biontech-covid-19--vaccine-what-you-need-to-know) | BNT162b2 | Aged 6 months and above | Two doses(aged 5 years and above); three doses(aged 6 months to 4 years) | Aged 12 years and above: 30 µg, 0.3 ml each, 4-8 weeks apart; aged 5 to 11 years: 10 µg, 0.2 ml each, 4-8 weeks apart, preferentially 8 weeks; aged 6 months to 4 years: 3 µg, 0.2 ml each, a schedule of two doses 3 weeks apart, a third dose at least 8 weeks. However, countries could consider extending the interval between the first and second dose up to 8 weeks; intramuscular-deltoid muscle |
| Inactivated viruses | [Sinopharm](https://www.who.int/news-room/feature-stories/detail/the-sinopharm-covid-19-vaccine-what-you-need-to-know) | COVID-19 vaccine BIBP | Aged 18 years and above | Two doses | 0.5 ml each dose, 3–4 weeks, intramuscular-deltoid muscle |
|  | [Sinovac](https://www.who.int/news-room/feature-stories/detail/the-sinovac-covid-19-vaccine-what-you-need-to-know) | CoronaVac | Aged 18 years and above | Two doses | 0.5 ml each dose, 2–4 weeks, intramuscular-deltoid muscle |
|  | [Bharat Biotech](https://www.who.int/news-room/feature-stories/detail/the-bharat-biotech-bbv152-covaxin-vaccine-against-covid-19-what-you-need-to-know) | BBV152 COVAXIN vaccine | Aged 18 years and above | Two doses | 0.5 ml each dose, 4 weeks, intramuscular-deltoid muscle |

**Supplementary Table 2 Search strategy**

| **PubMed**  (((COVID OR COVID-19 OR 2019-nCoV OR Coronavirus OR SARS-CoV-2) AND (vaccin* OR vaccine OR vaccination OR immunization)) AND ("Neoplasms"[Mesh] OR malign* OR malignant OR malignancy OR neoplasm* OR oncolog* OR oncology OR cancer OR carcinoma OR adenocarcinoma OR tumour OR tumor OR leukemia OR myeloma OR hepatoma OR lymphoma OR melanoma OR sarcoma OR blastoma OR fibrous histiocytoma OR neurofibrosarcoma OR neurilemmoma OR glioma OR glioblastoma OR medulloblastoma OR meningioma OR neuroblastoma OR chorio-epithelioma OR malignant mole OR seminoma OR dysgerminoma OR teratoma)) AND (review* OR systematic review OR meta OR meta-analysis OR meta-analyses OR meta-regression) |
| --- |
| **Cochrane library**  COVID OR nCoV OR Coronavirus OR SARS in All Text AND vaccin* OR Vaccine OR vaccination OR immunization in All Text AND cancer OR malign* OR malignant OR malignancy OR neoplasm* OR oncolog* OR oncology OR carcinoma OR adenocarcinoma OR tumour OR tumor OR leukemia OR myeloma OR hepatoma OR lymphoma OR melanoma OR sarcoma OR blastoma OR fibrous histiocytoma OR neurofibrosarcoma OR neurilemmoma OR glioma OR glioblastoma OR medulloblastoma OR meningioma OR neuroblastoma OR chorio-epithelioma OR malignant mole OR seminoma OR dysgerminoma OR teratoma in All Text AND review* OR systematic review OR meta OR meta-analysis OR meta-analyses OR meta-regression in All Text - (Word variations have been searched) |
| **Web of Science**  (((TS=(COVID OR COVID-19 OR 2019-nCoV OR Coronavirus OR SARS-CoV-2)) AND TS=(vaccine OR vaccination OR immunization)) AND TS=(cancer OR malignant OR malignancy OR neoplasm OR oncology OR carcinoma OR adenocarcinoma OR tumour OR tumor OR leukemia OR myeloma OR hepatoma OR lymphoma OR melanoma OR sarcoma OR blastoma OR fibrous histiocytoma OR neurofibrosarcoma OR neurilemmoma OR glioma OR glioblastoma OR medulloblastoma OR meningioma OR neuroblastoma OR chorio-epithelioma OR malignant mole OR seminoma OR dysgerminoma OR teratoma)) AND TS=(review OR systematic review OR meta OR meta-analysis OR meta-analyses OR meta-regression) |
| **EMBASE**  ((COVID or COVID-19 or 2019-nCoV or Coronavirus or SARS-CoV-2) and (vaccin* or Vaccine or vaccination or immunization) and (cancer or malign* or malignant or malignancy or neoplasm* or oncolog* or oncology or carcinoma or adenocarcinoma or tumour or tumor or leukemia or myeloma or hepatoma or lymphoma or melanoma or sarcoma or blastoma or fibrous histiocytoma or neurofibrosarcoma or neurilemmoma or glioma or glioblastoma or medulloblastoma or meningioma or neuroblastoma or chorio-epithelioma or malignant mole or seminoma or dysgerminoma or teratoma) and (review* or systematic review or meta or meta-analysis or meta-analyses or meta-regression)).af. |
| **CNKI**  SU=('COVID'+'COVID-19'+'2019-nCoV'+'Coronavirus'+'SARS-CoV-2') AND SU=('vaccine'+'vaccination'+'immunization') AND SU=('cancer'+'malignant'+'malignancy'+'neoplasm'+'oncology'+'carcinoma'+'adenocarcinoma'+'tumour'+'tumor'+'leukemia'+'myeloma'+'hepatoma'+'lymphoma'+'melanoma'+'sarcoma'+'blastoma'+'fibrous histiocytoma'+'neurofibrosarcoma'+'neurilemmoma'+'glioma'+'glioblastoma'+'medulloblastoma'+'meningioma'+'neuroblastoma'+'chorio-epithelioma'+'malignant mole'+'seminoma'+'dysgerminoma'+'teratoma') AND SU=('review'+'systematic review'+'meta'+'meta-analysis'+'meta-analyses'+'meta-regression') |

# Supplementary Table 3 Characteristics of included systematic reviews and meta-analyses

| **First author/**  **year** | **Search Details** | **No. of studies included; Meta-Analysis: Yes/No** | **Type of studies included** | **Study quality assessment** | **Outcome investigated** | **Cancer patients(range)** | **COVID-19 vaccine types** | **Cancer types** |
| --- | --- | --- | --- | --- | --- | --- | --- | --- |
| Abid, 2022 | 4 DBs (Medline, Scopus, Web of Science, and Cochrane databases), from 11 Dec. 2020 to 14 Sep. 2021. | 5 studies; No | Prospective and retrospective studies | NA | COVID-19 vaccine response | 70 (7-23) | BNT162b2, mRNA1273, JNJ-78436735 | Hematological malignancies |
| Becerril-Gaitan, 2022 | 3 DBs (PubMed, Cochrane Central Register of Controlled Trials, Web of Science), from 1st Mar. 2020 to 12th Aug. 2021 | 35 studies; Yes | Prospective and retrospective studies | ROBINS-I tool; 30 studies were moderate, 4 studies were serious and 1 study was critical risk of bias. | COVID-19 vaccine response | 2556 (24-315) | BNT162b2, mRNA1273, AZD1222, ChAdOx1, AD26.COV2.S, CoronaVac | Solid cancers, hematological malignancies |
| Cavanna, 2021 | 3 DBs (PubMed, Cochrane Library, and medRxiv up) until the date of 31 Jul 2021. | 6 studies; Yes | Observational prospective and cross sectional studies | NA | COVID-19 vaccine response and adverse events | 621 (24-200) | BNT162b2, mRNA-1273,  Ad26.COV2.S | Solid cancers, hematological malignancies |
| Corti, 2022 | 2 DBs (Medline (via PubMed) and Embase) carried out on 16  Aug. 2021. | 36 studies; No | Observational Cohort and Cross-Sectional Studies | NIH quality assessment tools; 2 studies "GOOD"(the least risk of bias), 34 studies “FAIR” (the study is susceptible to some bias deemed not sufficient to invalidate its results). | COVID-19 vaccine response and adverse events | 9260 (16-1503) | BNT162b2, MDN, OxA, Ad26.COV2.S, CoronaVac | Solid cancers, hematological malignancies |
| Gagelmann, 2021 | 3 DBs (MEDLINE, the Cochrane Library, and LitCovid); from 1 Jul. 2020 to 16 Sep.2021 | 49 studies; Yes | Observational Studies | A tool designed specifically to evaluate non-comparative studies; 39 studies were low and 10 studies were moderate risk of bias. | COVID-19 vaccine response and adverse events | 7322 (5-1445) | BNT162b2, mRNA-1273, AZD1222, Ad26.COV2.S | Solid cancers, hematological malignancies |
| Galmiche, 2022 | 2 DBs (Medline and Embase), from inception until 31 Aug. 2021. | 43 studies; No | Controlled interventional studies and observational cohorts | NIH quality assessment tools; 8 studies were of good quality, 24 studies were of fair quality, 10 studies were of poor quality, 1 study was with no quality assessment. | COVID-19 vaccine response and adverse events | 7739 (10-1445) | BNT162b2, mRNA-1273, Ad26.COV2.S, ChAdOx1, CoronaVac, NVX-CoV2373, BBV152-Covaxin, BBIBP-CorV | Solid cancers, hematological malignancies |
| Gong, 2021 | 2 DBs (PubMed and EMBASE), from 1 Dec. 2020 to 22 Jul. 2021 | 18 studies; Yes | NA | NA | COVID-19 vaccine response | 2834 | NA | Solid cancers, hematological malignancies |
| Gong, 2022 | 2 DBs (PubMed and EMBASE), from 1 Jan. 2021 to 4 Nov. 2021 | 64 studies; Yes | Cohort studies | Cohort studies and sub-studies of trials with a comparison group: NOS assessment tool, one-arm cohort studies: Joanna Briggs Institute Critical Appraisal Checklist for Cohort Studies. Cohort studies and sub-studies of trials with a comparison group: 8 studies were of good quality, 7 studies were of fair quality, 15 studies were of poor quality; one-arm cohort studies: 24 studies were low and 7 studies were high risk of bias. | COVID-19 vaccine response | 8546 (14-1455) | BNT162b2, mRNA-1273, Ad26.COV2.S, ChAdOx1 | Hematologic malignancies |
| Guven, 2022 | 1 DB (PubMed), from 1 Apr. 2021 to 4 Dec. 2021. | 26 studies; Yes | NA | NA | COVID-19 vaccine response | First dose: 996(30-276); second dose:3187(5-418) | Mostly mRNA vaccines | Hematologic malignancies |
| Guven, 2021 | 1 DB (PubMed), from 1 Apr. 2021 to 26 Jul. 2021. | 27 studies for qualitative synthesis, 17 studies for quantitative synthesis; Yes | Non-randomized trials | NOS; 14 studies were with ≧7 scores, 3 studies were with 6 scores. | COVID-19 vaccine response | First dose: 719(48-122); second dose: 1448(24-315) | BNT162b2, mRNA-1273, Ad26, AZD1222 | Solid cancers, hematological malignancies |
| Ito, 2022 | 2 DBs (PubMed and WHO COVID-19 database), up to 2 Oct. 2021. | 30 studies; Yes | Non-randomized trials | NOS; NA | COVID-19 vaccine response | 2203 (18-276) | BNT162b2, mRNA-1273, Ad26.COV2.S,ChAdOx1, AZD1222 | Lymphoid malignancy |
| Lee, 2022 | 5 DBs (PubMed, Embase, Central Register of Controlled Trials, COVID-19 Open Research Dataset Challenge (CORD-19), and WHO COVID-19 databases),from 1 Dec. 2020 to 5 Nov. 2021. | 18 studies on solid cancers, 21 studies on hematological cancers; Yes | Prospective  observational studies | ROBINS-I tool; solid cancers: 13 studies were low and 5 studies were moderate risk of bias; hematological cancers: 18 studies were low and 3 studies were moderate risk of bias. | COVID-19 vaccine response | First dose: 1950 (18-267); second dose: 3924 (5-529) | BNT162b2, mRNA-1273, AZD1222 | Solid cancers, hematological malignancies |
| Marra, 2022 | 6 DBs (PubMed, Cumulative Index to Nursing and Allied Health (CINAHL), EMBASE, Cochrane Central Register of Controlled Trials, Scopus, and Web of Science), from 1 Dec. 2019 to 10 Aug. 2021. | 8 studies; Yes | Prospective cohort, retrospective cohort studies | Downs and Black scale; 5 studies were of good quality, 3 studies were of fair quality. | COVID-19 vaccine response and effectiveness | 1853 (36-857) | BNT162b2, mRNA-1273, AZD1222, Coronavac | Solid cancers, hematological malignancies |
| Mehrabi Nejad,2022 | 3 DBs (PubMed-MEDLINE, Scopus, and Web of Science), from Jun 1, 2020 to Sep. 1, 2021. | 9 studies; Yes | Cross-sectional, Prospective  cohort and Retrospective  cohort | NIH quality assessment tool; 7 studies were of good quality, 2 studies were of fair quality. | COVID-19 vaccine response | 1293 (36-315) | BNT162b2 | Solid cancers, hematological malignancies |
| Molica, 2022 | 1 DB (PubMed), before 5 Nov.2021 | 13 studies; Yes | NA | NA | COVID-19 vaccine response | 2082 | BNT162b2, mRNA-1273, ChAdOx1 | Chronic lymphocytic leukemia |
| Sakuraba, 2022 | 3 DBs (PubMed/MEDLINE, EMBASE, and medRxiv); from inception to Aug. 1, 2021 | 16 studies; Yes | Observational studies | Joanna Briggs Institute Critical Appraisal Checklist/ GRADE approach; A majority of the studies were of medium to high quality. | COVID-19 vaccine response | 1453 | BNT162b2, mRNA-1273 | Solid cancers, hematological malignancies |
| Schietzel, 2022 | 4 DBs (PubMed, Embase, Medrxiv and SSRN); up to 21 Aug. 2021 | 7 studies; Yes | Non- randomized studies | NOS; Risk of bias ratings for the included studies were low to moderate. | COVID-19 vaccine response | 601 (23-143) | BNT162b2, mRNA-1273, Ad26.COV2.S | Hematological malignancies |
| Teh, 2022 | 3DBs ((MEDLINE, EMBASE, and Cochrane CENTRAL); from 1 Jan. 2021 to 31 Aug. 2021 | 44 studies; Yes | Prospective cohort studies, retrospective  cohort studies | NOS; 23 studies (52%) were evaluated as being of good or fair quality (good, 11; fair, 12) with low risk of bias, whereas the remaining 21 studies were rated as poor quality (high risk of bias) | COVID-19 vaccine response and adverse events | First dose: 2331 (13-685); second dose: 4733(113-1446) | BNT162b2, mRNA-1273, ChAdOx1, Ad26, ND | Hematological malignancies |

ROBINS-I: Risk Of Bias In Non-randomized Studies of Interventions; NIH: National Institutes of Health; NOS: Newcastle-Ottawa Scale; GRADE: Grades of Recommendation, Assessment, Development and Evaluation
